# Role of Multimodal Analgesia in the Evolving Enhanced Recovery after Surgery Pathways

**DOI:** 10.3390/medicina54020020

**Published:** 2018-04-23

**Authors:** David Gelman, Arūnas Gelmanas, Dalia Urbanaitė, Ramūnas Tamošiūnas, Saulius Sadauskas, Diana Bilskienė, Albinas Naudžiūnas, Edmundas Širvinskas, Rimantas Benetis, Andrius Macas

**Affiliations:** 1 Department of Cardiac, Thoracic and Vascular Surgery, Medical Academy Lithuanian University of Health Sciences, Kaunas 50009, Lithuania; gelmanas@gmail.com (D.G.); sirdiskr@lsmuni.lt (E.Š.); rimantas.benetis@kaunoklinikos.lt (R.B.); 2 Department of Anaesthesiology, Medical Academy, Lithuanian University of Health Sciences, Kaunas 50009, Lithuania; dalia.urbanaite@gmail.com (D.U.); ramunas.tamosiunas@lsmuni.lt (R.T.); diana.bilskiene@kaunoklinikos.lt (D.B.); andrius.macas@lsmuni.lt (A.M.); 3 Department of Internal Diseases, Medical Academy, Lithuanian University of Health Sciences, Kaunas 50009, Lithuania; saulius.sadauskas@fc.lsmuni.lt (S.S.); albinas.naudziunas@lsmuni.lt (A.N.)

**Keywords:** enhanced recovery after surgery, multimodal analgesia, postoperative pain, NSAIDs

## Abstract

Enhanced recovery after surgery (ERAS) are specially designed multimodal perioperative care pathways which are intended to attain and improve rapid recovery after surgical interventions by supporting preoperative organ function and attenuating the stress response caused by surgical trauma, allowing patients to get back to normal activities as soon as possible. Evidence-based protocols are prepared and published to implement the conception of ERAS. Although they vary amongst health care institutions, the main three elements (preoperative, perioperative, and postoperative components) remain the cornerstones. Postoperative pain influences the quality and length of the postoperative recovery period, and later, the quality of life. Therefore, the optimal postoperative pain management (PPM) applying multimodal analgesia (MA) is one of the most important components of ERAS. The main purpose of this article is to discuss the concept of MA in PPM, particularly reviewing the use of opioid-sparing measures such as paracetamol, nonsteroid anti-inflammatory drugs (NSAIDs), other adjuvants, and regional techniques.

## 1. Introduction

Enhanced recovery after surgery (ERAS) are specially designed multimodal perioperative care pathways which are intended to attain rapid recovery after major surgery by supporting preoperative organ function and attenuating the stress response caused by surgical trauma [1,2,3,4,5]. The conception of cardinal changes to and improvements in various elements of hospital healthcare systems was implemented by Professor Henrik Kehlet in Copenhagen in the 1990s [1]. This resulted in setting up the investigation group which prepared and published the very first evidence-based ERAS protocol for patients undergoing colonic surgery in 2005 [1,6]. Since the first publication, the ERAS protocol has been modified and adapted based on data from evidence-based trials, across various surgical disciplines, for example,, for colorectal surgery [7], spine surgery, orthopaedic surgery, radical cystectomy [8], and gynaecologic/oncology surgeries [9]. The main philosophy of the ERAS protocol is the multimodal approach involving three major elements—preoperative, perioperative, and postoperative components—which support and accelerate the return of functions that allow patients to get back to normal activities rapidly [1,2]. For successful implementation of the enhanced recovery protocol (ERP), a multidisciplinary collaboration between patients, surgeons, anaesthetists, physiotherapists, occupational therapists, and nursing staff is a necessary condition [2,10]. Although ERPs vary amongst healthcare institutions, the earlier-mentioned key elements remain the cornerstones [11]. Being aware that optimal postoperative pain management (PPM) may lead to rapid recovery, better outcomes, and shorter length of hospital stay (LOHS) after major surgery, it remains a real challenge to medical staff to control postoperative pain effectively by applying multimodal analgesia [11,12]. The main purpose of this article is to discuss the concept of MA in PPM, particularly reviewing the use of opioid-sparing measures such as paracetamol, nonsteroid anti-inflammatory drugs (NSAIDs), other adjuvants, and regional techniques.

## 2. Postoperative Pain Management (PPM)

Postoperative pain (PP) influences the quality and length of patients’ postoperative recovery period, and later, the quality of life [11,12]. One of the principal components of ERAS is the good control of PP, and multimodal analgesia (MA) seems to be the optimal modality for this [11]. Assessment of PP is a major challenge requiring the ability to use the professional’s accuracy and objectivity [12]. PPM is closely bound with the provision of information to the patient about the PP control before surgery is performed. Therefore, it is necessary to form a pain assessment and treatment plan together with the patient. The selection of pain assessment tool must be considered, and the patient must be trained to use it properly in order to facilitate the PPM; that is, using the numeric rating system (NRS) or the visual analogue scale (VAS) [12] as the tool. Setting the pain level above which analgesia must be adjusted or other analgesic technique must be selected is the necessity.

## 3. Epidural Analgesia (EDA)

From the beginning of the ERAS era, EDA was considered as optimal PPM method and was integrated into most ERAS protocols involving thoraco-abdominal and lower limb surgery [8,13,14,15,16,17,18]. According to clinical trials, it is recommended to apply continuous EDA (with opioids or without them) after open abdominal surgeries, for better pain relief and fewer complications in the respiratory system, compared with the usage of intravenous opioids, are reported [19]. Equally, EDA results in the earlier return of bowel function compared with patient-controlled analgesia (PCA) as well as attenuated stress response and insulin resistance and reduced incidence of respiratory and cardiovascular complications in colorectal surgery [1].

However, Hübner et al. [20], according to their small study, suggest that EDA affects haemodynamics and that transitory haemodynamic support is essential. This may manifest due to the sympathetic trunk block [19]. Khan et al. [16] claims that thoracic EDA improved the return of bowel function. Hence, EDA interferes with recovery after laparoscopic colorectal surgery. Yet, the hospital stay remains unchanged [16,20]. Some authors [17,18] claim that EDA usage in orthopaedic surgeries, e.g., total knee arthroplasty, prolongs postoperative recovery after surgery and causes motor blockage that manifests as knee buckling, and later, perambulation. There is a small risk of epidural haematoma and epidural abscess as well [21,22,23,24]. Therefore, the role of EDA in the ERAS setting has been questioned recently, and there was a global call for evidence-based alternative PPM methods with comparable analgesia to EDA, but without its adverse effects. Table 1 outlines some important trials supporting the use of MA as an alternative to EDA. 

## 4. Multimodal Analgesia

Use of more than one analgesic modality has become the necessity in order to achieve effective PP control [11,30]. Systemic administration of two or more drugs that are strategically combined to block pain perception at various locations in the peripheral and central nervous systems for providing analgesia may improve pain relief and reduce opioid consumption [3,31,32]. Regional methods, for instance, the transversus abdominis plane (TAP) block, rectus sheath block, paravertebral block (PVB), other nerve blocks, or continuous wound infiltration (CWI) are integral parts of MA as well and provide further reduction of systemic analgesic doses [20,25,26,27,28,29].

MA enables better analgesia with fewer side effects and quicker postoperative recovery, which may be related to better results and outcomes [11,12]. Postoperative MA may consist of the use of opioid or nonopioid (i.e., nonsteroid anti-inflammatory drugs (NSAIDs) with acetaminophen) pharmacological agents with additional regional anaesthesia. Evidence strongly supports the usage of the TAP block in abdominal surgery, which significantly reduces PP and opioid consumption, though the result is obvious only during the first two days after surgery [19]. Addition of the bilateral TAP block performed in laparoscopic colorectal surgery was found to be effective in the reduction of postoperative opioid consumption and LOHS [3]. Continuous intra-articular infusion through wound catheters and local intra-articular infiltration after orthopaedic interventions, for instance, arthroscopic, total hip, or knee replacement surgeries, reduces PP and opioid consumption and shortens LOHS [3,17,18,32].

Intravenous lidocaine (IL) is another agent found in ERAS protocols. IL has analgesic, antihyperalgesic, and anti-inflammatory effects, therefore it seems to be an ideal medication for pain control [33]. According to McCarthy et al. [34], IL used in the perioperative period was noticed as having a highly positive effect for PPM (lower pain scores after surgery, reduced postoperative analgesic requirements) and the duration of ileus, LOHS, nausea, and vomiting in patients undergoing abdominal surgery. Naik et al. [33] suggest that IL used perioperatively and continued postoperatively (according to approved protocols) also has benefits on pain scores and opioid use in patients undergoing colorectal surgery.

Ketamine is reported to have analgesic and antihyperalgesic properties when administered through the intravenous route. In their study, Kaur et al. [35] found that an intraoperative infusion of low-dose ketamine (0.1 mg/kg/h following bolus 0.2 mg/kg) during open cholecystectomy under general anaesthesia caused effective analgesia in the first six hours of postoperative period and statistically significant reduced the consumption of opioids in the first 24 h after surgery (*p* = 0.001); only five patients (12.5%) required rescue analgesic. However, ketamine remains a controversial agent due to adverse effects; therefore, it is essential to apply the appropriate dose.

Gabapentinoids (gabapentin or pregabalin) has been used as an adjunct for PPM in orthopaedics and major gynaecological surgery frequently [36,37,38]. Peng et al. [39] found a high dose (>900 mg) of gabapentin to be more effective in reducing PP after spine surgery. However, a meta-analysis [36] revealed no difference in pain scores after total knee arthroplasty between patients provided with the gabapentinoid class of drugs and a placebo.

Opioids remain the mainstay of postoperative analgesia for moderate to severe pain [11,12,31]. However, it is important not to forget dose-related short-term and long-term side effects, including nausea and vomiting, urinary retention, intestinal obstruction, pruritus, and respiratory depression [1,11,12,31]. Many patients suffer from chronic pain, wherefore the long-term opioid usage increases the risk of experiencing stronger postoperative pain, increased opioid need, and prolonged postoperative recovery period and LOHS, and may have an influence on central and peripheral sensitization and impact the development of abnormally heightened sensitivity to pain [12,31,40]. 

Hence, the effort has been made to modify multimodal regimens in ERAS protocols that reduce opioid demand and opioid-related side effects. These drugs are less than ideal after abdominal surgery [30]. Thus, opioids are generally used as rescue analgesics in the case of insufficient PPM by using nonopioid medications [30,31].

## 5. Nonsteroidal Anti-Inflammatory Drugs (NSAIDs) and Paracetamol (Acetaminophen)

NSAIDs block both cyclooxygenase isoforms (COX-1, COX-2) competitively, which results in the inhibition of the synthesis of prostaglandins and thromboxanes [41]. The anti-inflammatory, analgesic, and antipyretic effects are achieved by inhibiting COX-2, while the increased risk of postoperative bleeding, gastrointestinal tract ulcers, and renal dysfunction is related to inhibition of the COX-1 isoform [42,43]. Furthermore, it is claimed that NSAIDs are not recommended for patients with concomitant cardiovascular pathology due to undesirable side effects. Long-term, high-dosage use of NSAIDs is associated with increased risk of cardiovascular events such as myocardial infarction (MI) or stroke [44,45,46]. In their study, Olsen et al. [47] revealed that even short-term administration of NSAIDs was associated with increased risk of death and recurrent MI in patients with prior MI. Nevertheless, it is claimed that these analgesics, used in multimodal regimens in appropriate doses, improve pain scores and reduce narcotic requirements and unwanted effects related to opioids [11,12,31,48].

Sharma et al. [49] reviewed twenty-five trials that analysed the effects of NSAIDs or acetaminophen and opioid intake on PP in spinal surgery. They reported that the vast majority of these studies showed that these analgesics improved PP management significantly at the first and second postoperative days and reduced postoperative morphine consumption.

Amin et al. [50] claimed that the administration of NSAIDs in combination either with local anaesthetic or with morphine in arthroscopic knee surgery has = synergistic action whereby the inflammatory response caused by surgical trauma decreases, which results in reduced pain, shorter LOHS, and faster rehabilitation. In their study, the intra-articular analgesic effects of ropivacaine and morphine with or without lornoxicam and the need for rescue intravenous morphine at rest and during movement was compared in forty-five patients after anterior cruciate ligament reconstruction under spinal anaesthesia. The addition of lornoxicam resulted in significantly lower pain scores (using VAS and The Western Ontario and McMaster Universities Arthritis Index (WOMAC) scores), reduced opioid consumption, reduced occurrence of side effects, reduced LOHS, and patients were also able to start walking after surgical interventions earlier. Lornoxicam, introduced to the market in the 1990s, is one of the NSAIDs described as being as potent as narcotics (i.e., morphine, pethidine (meperidine), and tramadol) in relieving PP following gynaecological or orthopaedic interventions, and its effectiveness is comparable with other NSAIDs after oral surgery [50,51,52]. It is more potent than any other NSAIDs; well-tolerated, especially the parenteral form [50]; and using this medication in combination with other NSAIDs may be considered as a better alternative or adjuvant to narcotics to control moderate and severe pain [51,52].

Concerns have recently been raised about the relation between anastomotic leakage and NSAIDs in gastrointestinal surgery [53]. Recent meta-analysis of six randomised controlled trial (RCTs) showed an increased risk of anastomotic leak; however, it was not significant (Peto odds ratio (OR) 2.16 (95% confidence interval (CI) 0.85–5.53, *p* = 0.11)) [54]. The effect of Lornoxicam on the integrity of colonic anastomosis was evaluated in rats, and again, the anastomotic leak risk was increased insignificantly [55]. Overall, the proof is inconclusive and does not encourage the avoidance of short perioperative NSAID treatment in the ERAS setting [56].

Acetaminophen is an effective medication in reducing both mild and moderate pain, it is claimed to have a highly favourable safety profile, and its harmful effects are quite uncommon (nausea and vomiting, skin irritation, thrombocytopenia, liver enlargement) [11,31]. According to certain authors, intravenous paracetamol may also reduce PP in patients experiencing orthopaedic and spinal surgery [12,57]. Rosero and Joshi claim that its effectiveness can be highly intensified by using suitable doses and using it in combination with NSAID or COX-2 inhibitors [31]. For example, the combination of single-dose paracetamol (0.5–1 g) and ibuprofen (200–400 mg) after dental surgery induce superior postoperative pain relief to that of each drug used separately [11].

In their review, Ong et al. [58] evaluated the efficacy of the combination of paracetamol and an NSAID versus either drug alone in various acute pain models on the basis of twenty-one clinical studies. They found that the combination of paracetamol and an NSAID may provide superior analgesia to that of either drug alone in 85% and 64% of the studies, respectively.

In the systematic Cohrane review [59], it is claimed that the combination of fixed doses of oral ibuprofen and paracetamol provides a superior analgesic effect to that of using each medication alone at the same doses, and lessens likelihood of the need of additional analgesic over about eight hours in PPM.

Nelson et al. [9] claims that paracetamol and NSAIDs in combination should be administered regularly to all patients after gynaecologic/oncology surgery unless contraindication exists and it is a high level of evidence recommendation.

## 6. Conclusions

ERAS protocols are intended to improve and facilitate faster recovery after surgery. However, the implementation of this goal is a real challenge to medical staff. Acute postoperative pain is one of the major causes that affects recovery after surgery. Therefore, sufficient postoperative pain control is essential to improve the quality of convalescence and speed it up. The role of EDA as a gold standard in thoraco-abdominal and lower limb surgery in the ERAS setting has been questioned lately, as its side effects such as haemodynamic instability, motor block, and urinary retention could impair fast recovery after surgical procedures. Consequently, choosing multimodal analgesia, including such analgesics as paracetamol and nonsteroidal anti-inflammatory drugs in combination with regional nerve blocks or wound infiltration, is claimed to provide comparable postoperative analgesia to EDA, reduce opioid requirements, and provide faster recovery and improved patient satisfaction after surgical procedures.

## Figures and Tables

**Table 1:** Evidence-based alternatives to epidural analgesia in the setting of ERAS.

Type of Surgery	Preoperative Analgesia	Intraoperative Analgesia	Postoperative Analgesia
Open colorectal cancer surgery Bertoglio et al., 2012 [25]	None	Fentanyl 2–5 mcg/kg/h	Ropivacaine 0.2% 10 mL/h through preperitoneal catheter (above the peritoneum within the musculofascial layer) Ketolorac 30 mg × 3 IV Acetaminophen 1 g × 4 PO
Open gynaecology/oncology surgery Kalogera et al., 2013 [26]	Celecoxib 400 mg × 1 PO Acetaminophen 1 g × 1 PO Gabapentin 600 mg × 1 PO	Opioids IV at discretion of anesthesiologist supplemented with ketamine, ketorolac, or both. After incision closure: injection of bupivacaine at incision site	Oxycodone 5–10 mg as required PO, max 6 doses/day Acetaminophen 1 g × 4 PO Ketorolac 15 mg × 4 IV on Day 1, then Ibuprofen 800 mg × 4 PO Hydromorphone for rescue analgesia
Primary total knee arthroplasty McDonald et al., 2011 [27]	2 h before surgery: Temazepam 10–20 mg × 1 Dexamethasone 10 mg × 1 Gabapentin 300 mg × 1 Acetaminophen 1 g × 1	Spinal anesthesia with 2.75–3.2 mL 0.5% Bupivacaine. 200 mL intra-articular 0.2% Ropivacaine (at the end of surgery).	Gabapentin 300 mg × 2 Acetaminophen 1 g × 4 Ibuprofen 400 mg × 3 Oxycodone 5–10 mg 2–4 hourly as required Three bolus doses of 40 mL Ropivacaine (0.2%), each via intra-articular catheter at 4 h post-surgery, 2300 h, and 0800 h the following morning. Intra-articular catheter then removed.
Hip hemiarthoplasty for fractured neck of femur Talboys et al., 2015 [28]	Preoperatively, patients are prescribed a dose of acetaminophen of 1 gram (g) PO and tramadol M/R 50–100 mg × 2. A fascia iliaca compartment block (FICB) comprising of 30 mL of levobupivacaine 0.25% is given in the emergency department.	A single shot of spinal bupivacaine (2.5–3.0 mL); no intrathecal opiates are used.IV dexamethasone 8 mg and diclofenac 75 mg. Perioperatively, the surgeon infiltrates the joint with 150–200 mL of ropivicaine 0.2%. A periarticular catheter is then set up to deliver an infusion of the LA: an initial 20 mL bolus followed by an infusion rate of 8 mL/h (with 5 mL bolus every 20 min).	Gabapentin 300 mg twice daily for 5 days Acetaminophen 1 g × 4 Ibuprofen 400 mg × 3 for 1 week When required, tramadol M/R 50–100 mg × 2 Severe breakthrough pain is managed with Oramorph liquid 5–10 mg every 2 hours.
Open abdominal aortic surgery Renghi et al., 2013 [29]	Proparacetamol 2 g × 1 Fentanyl 100 mcg × 1	Fascia of the parietal peritoneum was infiltrated subcutaneously with 20 mL of levobupivacaine (0.5%)	At the end of surgery, subfascial and subcutaneous placement of a double-multiperforated catheter was performed, and an infusion of levobupivacaine, 0.25% at 4 mL/h, was started. Ibuprofen 600 mg × 3 PO Ketorolac 30 mg IV for rescue analgesia
Laparoscopic colorectal surgery Hubner et al., 2015 [20]	None	Fentanyl at discretion of anesthesiologist	Morphine PCA Paracetamol 1 g × 4 PO Metamizole 500 mg × 4 PO

Abbreviations: IV—intravenous; PO—per oral; LA—local anaesthetic; M/R—modified release; PCA—patient controlled analgesia.
